# Supplementary figures and images for: NK cell-induced damage to P.falciparum-infected erythrocytes requires ligand-specific recognition and releases parasitophorous vacuoles that are phagocytosed by monocytes in the presence of immune IgG
Source: PLoS Pathog. 2023 Nov 8;19(11):e1011585. doi: 10.1371/journal.ppat.1011585 (PMC10659167; doi:10.1371/journal.ppat.1011585)

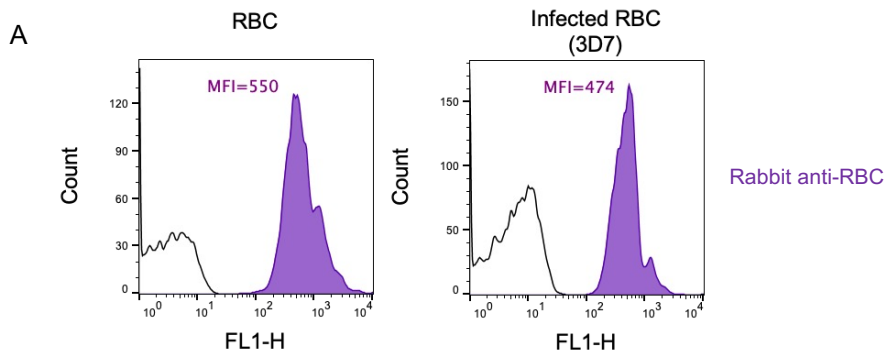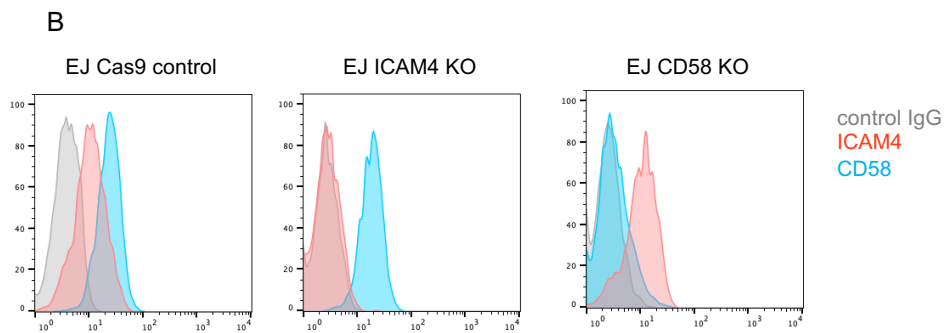

Supplement: S1 Fig — (A) Flow cytometry profiles of cell surface staining using Rabbit anti-RBC polyclonal antibody in uninfected and 3D7-infected RBC. (B) Flow cytometry profiles of cell surface staining of Cas9 control, ICAM4 KO and CD58 KO EJ cells for ICAM4 and CD58, showing the loss of ICAM4 and CD58 in the respective knockout cells. (PDF) [file ppat.1011585.s001.pdf]

A

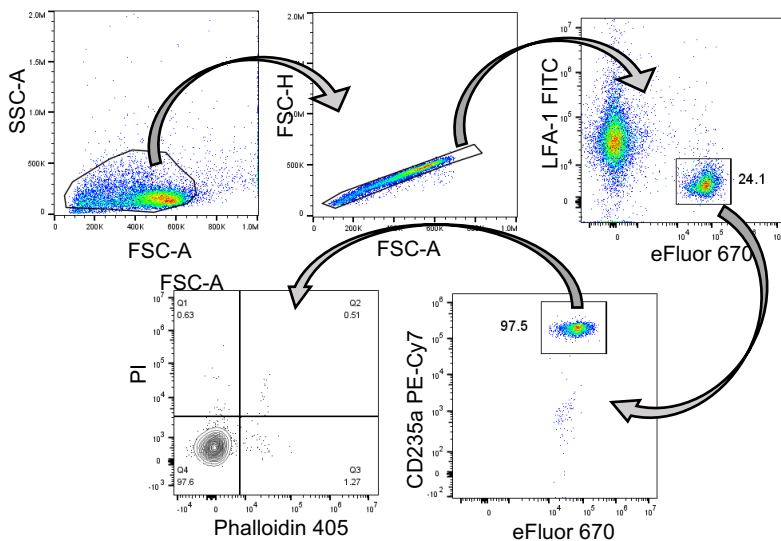

B

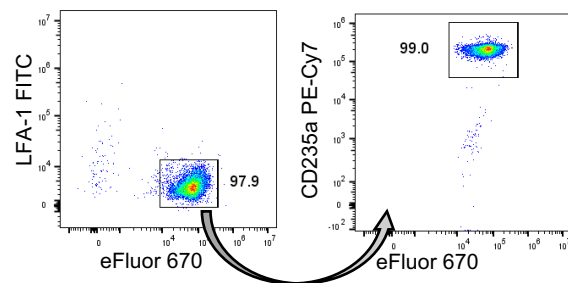

C

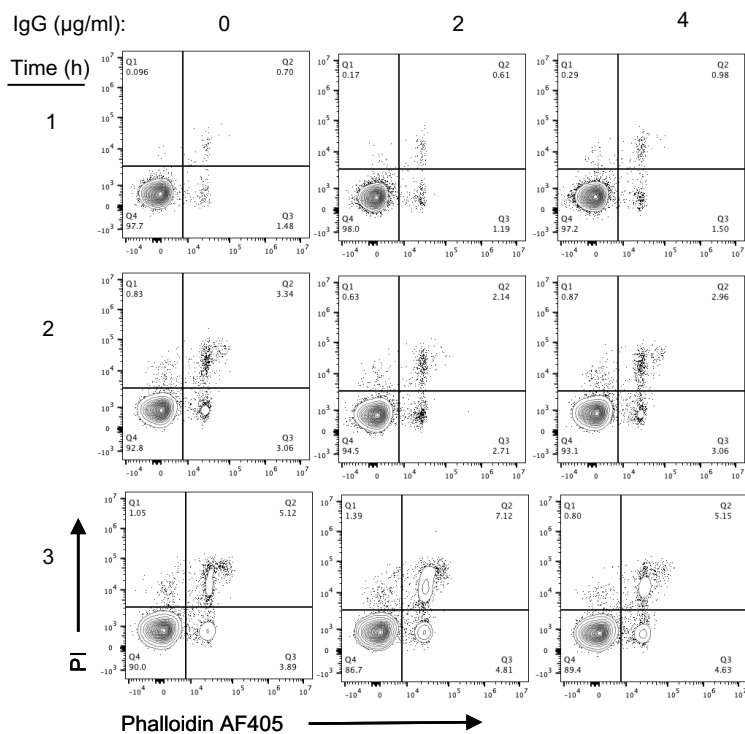

Supplement: S2 Fig — (A) Gating of eFluor 670+ glycophorin A (CD235a)+ iRBC and LFA-1+ NK cells to determine damage to iRBC with Phalloidin-AF405 and to the parasite with PI. (B) Same analysis as in (A) of iRBC incubated for 3 hours with anti-RBC IgG in the absence of NK cells. (C) Representative experiment performed as in Fig 2C performed in the absence of NK cells. (PDF) [file ppat.1011585.s002.pdf]

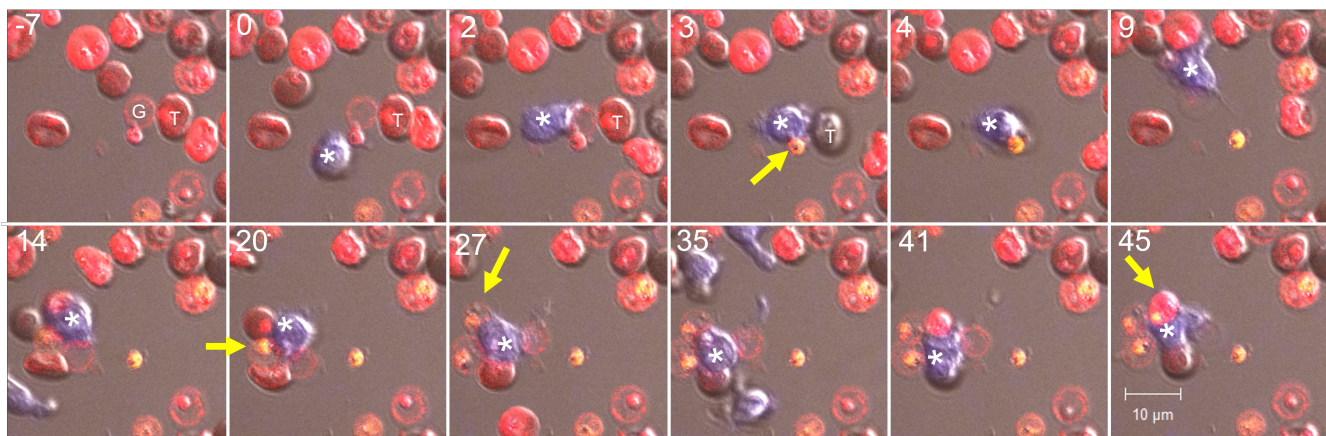

Supplement: S3 Fig — The experimental setup is identical to that shown in Fig 3. eFluor 450-stained primary NK cells (blue) were incubated with eFluor 670-stained iRBC (red) at a ratio of 1:3 in the presence 2 μg/ml anti-RBC IgG. PI was added at the start of image recording. The 12 time frames span 52 minutes. Numbers at the upper left corner of each panel indicate the time in minutes relative to the first NK–iRBC contact, which was set as minute 0. A ghost iRBC (labeled G in the first panel, -7 minutes) was contacted by an NK cell (marked with an asterisk) at time 0. This ghost iRBC included a PI-negative PV and was adjacent to an intact iRBC target (marked T). Activation of the NK cell in contact with the iRBC ghost was detected after 2 minutes (t = 2) through enhanced motility and plasma membrane extensions. PV damage was observed 3 minutes later (yellow arrow). One minute later (t = 4 min), the NK cell covered the iRBC target and began to move away. The ghost iRBC membrane was dragged away by the NK cell, leaving a free PI+ PV behind (t = 9). The motile NK cell contacted several other iRBC (beginning at t = 9). PI+ PV appeared at t = 20, 27, and 45 (yellow arrows). Note the presence of a PV in a ghost iRBC (bottom right) that remained PI-negative for at least 52 minutes. (PDF) [file ppat.1011585.s003.pdf]

A

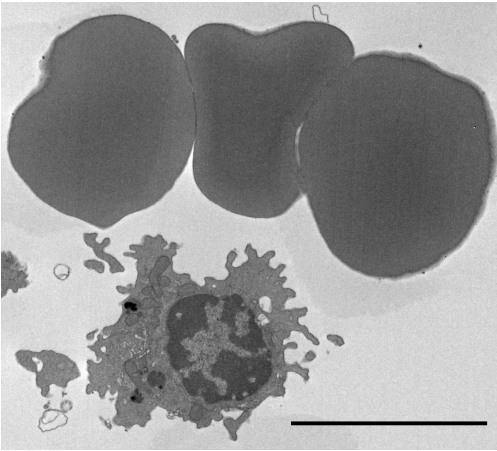

B

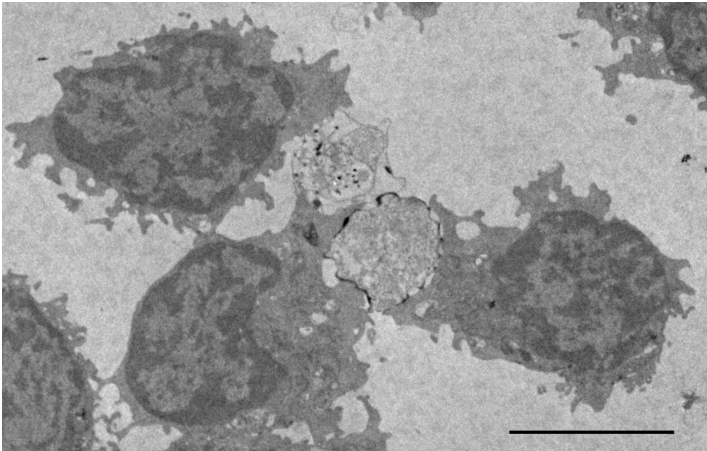

C

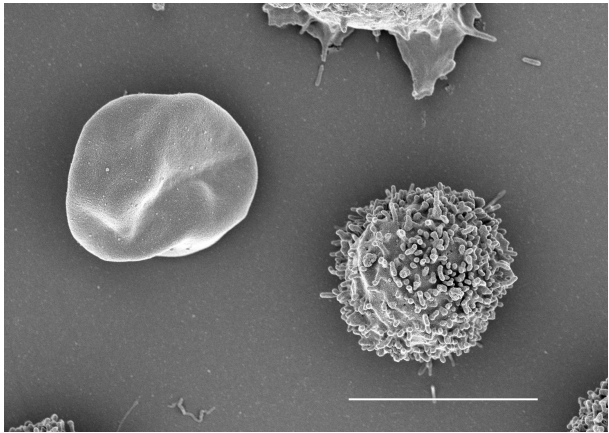

D

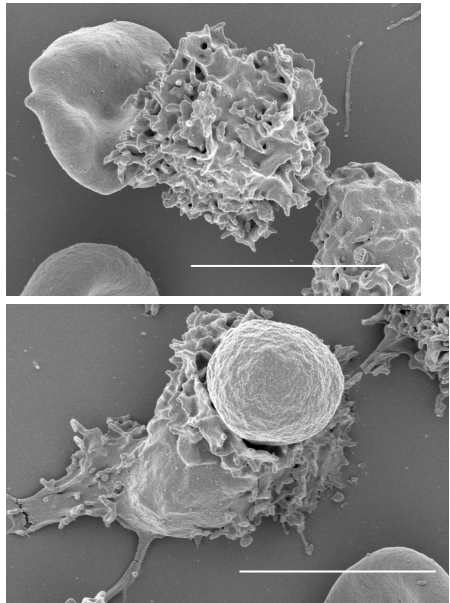

E

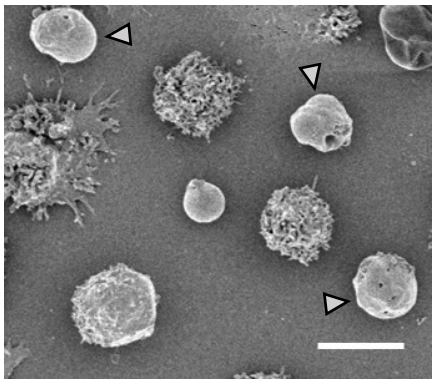

Supplement: S4 Fig — (A) TEM image of an NK cell and three uninfected RBC, which had been co-incubated in the presence of 2 μg/ml anti-RBC antibody for 3 hours. Scale bar is 5 μm. (B) TEM image of three NK cells in contact with two iRBC during incubation with 2 μg/ml anti-RBC antibody. Two NK cells (lower left and lower right) that flank the same iRBC were polarized, as seen by nucleus positioned at the back of the cell, away from the immunological synapse with the iRBC target. The lower left NK cell formed synapses with 2 iRBC. The NK cell at the top, apparently less polarized, may have been detaching from a ghost iRBC. Scale bar is 5 μm. (C) SEM image of an NK cell and an iRBC co-incubated in the absence of anti-RBC antibody. Scale bar is 5 μm. (D) SEM images of NK cells in contact with iRBC after incubation with 2 μg/ml anti-RBC antibody. The iRBC in the first image (top panel) appears to have retained some rigidity. In the lower panel, loss of iRBC rigidity suggests that damage by the NK cell had occurred. Scale bars are 5 μm. (E) SEM image of a PV at the center, which was surrounded by four NK cells and three iRBC ghosts (arrowheads), taken from a sample obtained after coincubation of NK cells and iRBC in the presence of 2 μg/ml anti-RBC antibody. Scale bar is 5 μm. (PDF) [file ppat.1011585.s004.pdf]

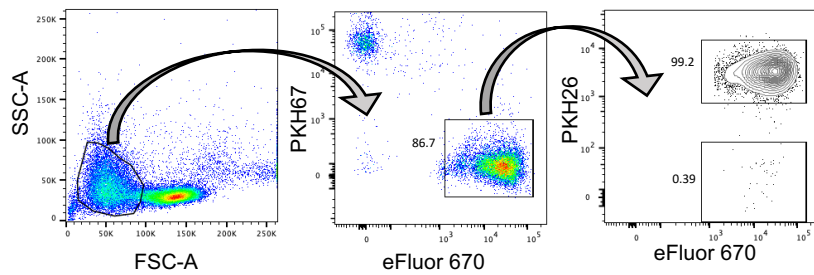

Supplement: S5 Fig — EXP2-GFP+ iRBC were stained with eFluor 670 and membrane dye PKH26. NK cells were stained with PKH67. iRBC were gated away from NK cells first by forward and side scatter, and then gated for eFluor670+ PKH67-negative cells, followed by gating the PKH26-negative eFluor670+ population consisting of free PV. (PDF) [file ppat.1011585.s005.pdf]

A

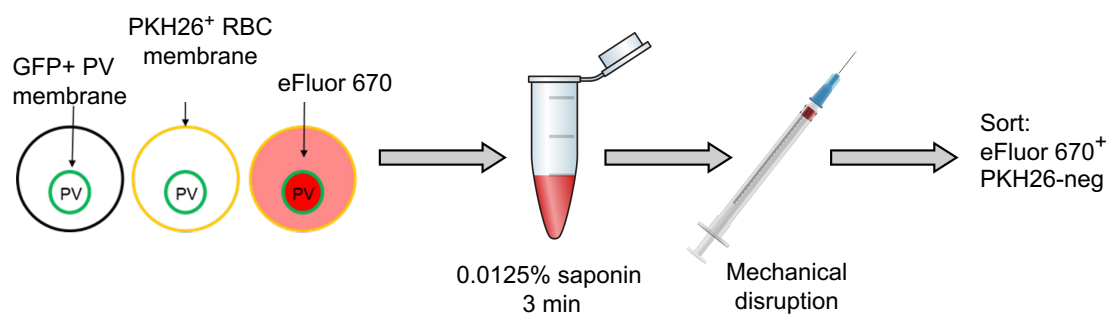

B

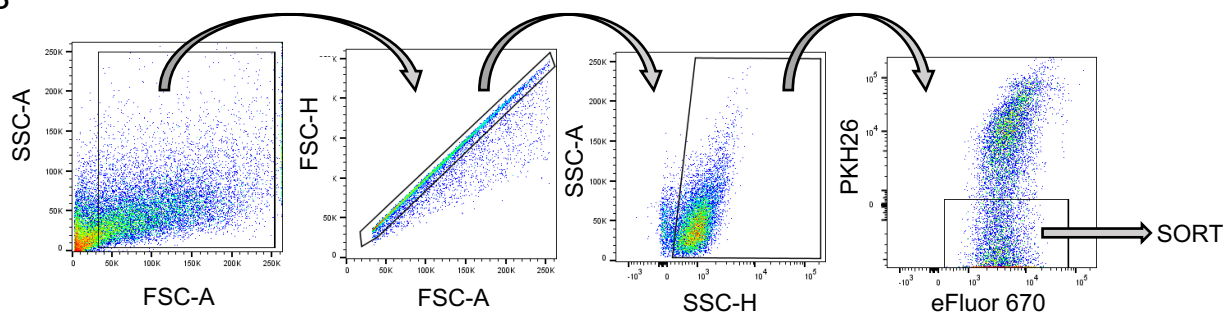

C

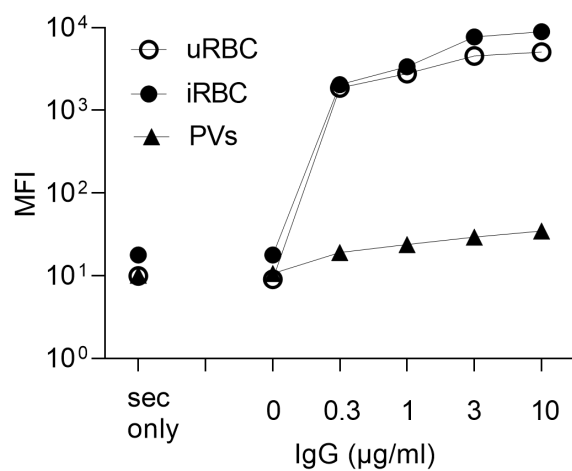

Supplement: S6 Fig — (A) RBC infected with 3D7 PfEXP2-GFP were stained with membrane dye PKH26 and eFluor 670 and treated with 0.0125% saponin for 3 minutes, followed by a wash and mechanical disruption of the iRBC plasma membrane through a 31-gauge needle. (B) Samples were sorted by flow cytometry for PKH26-negative eFluor 670+ particles. (C) Mean fluorescence intensity (MFI) of uninfected RBC (uninfected RBC), iRBC, and PV stained with different concentrations of anti-RBC IgG. The first set of points represent samples stained with secondary Ab only (sec only). (PDF) [file ppat.1011585.s006.pdf]

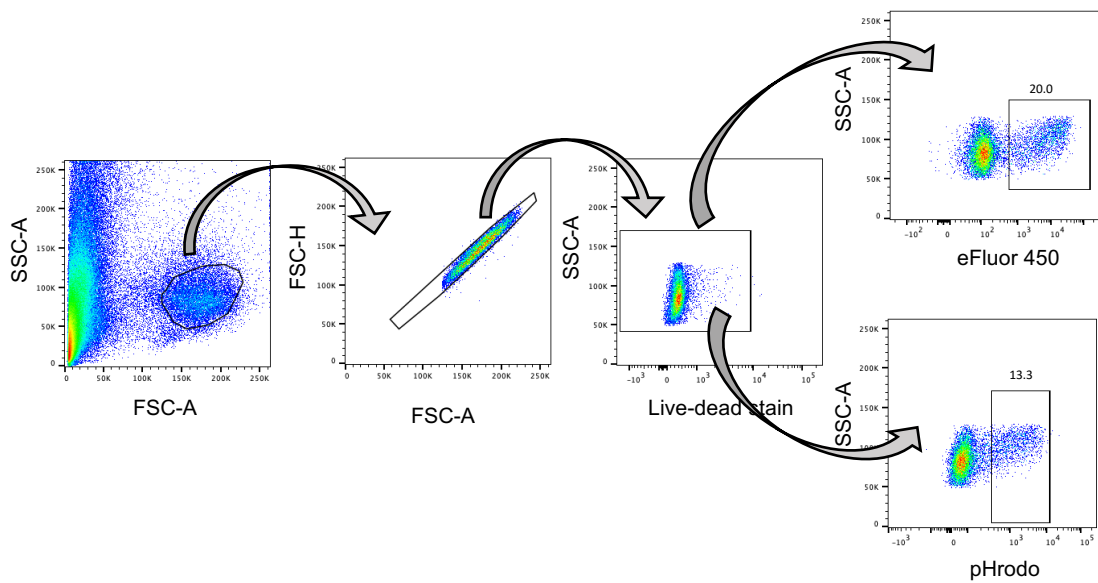

Supplement: S7 Fig — Monocytes previously incubated with PV were stained with live-dead stain and gated for live monocytes and further gated for either eFluor 450+ or pHrodo+ monocytes. (PDF) [file ppat.1011585.s007.pdf]
