# Supplementary material for: NK cell-induced damage to P.falciparum-infected erythrocytes requires ligand-specific recognition and releases parasitophorous vacuoles that are phagocytosed by monocytes in the presence of immune IgG
Source: PLoS Pathog. 2023 Nov 8;19(11):e1011585. doi: 10.1371/journal.ppat.1011585 (PMC10659167; doi:10.1371/journal.ppat.1011585)
Supplement: Attachment — Submitted filename: PLoS_response to reviewers_101123.pdf [file ppat.1011585.s014.pdf]

Part I - Summary
Please use this section to discuss strengths/weaknesses of study, novelty/significance, general execution and scholarship.

Reviewer #1: In this beautifully written paper by Sekar et al, the authors demonstrate how NK cells are able to both bind opsonized Pf-infected RBCs and what happens to parasitic contents following NK-cell mediated ADCC. In a series of elegant experiments combining both antibody blocking experiments and gene ablation in erythrocyte precursors, the authors show that red cell expression of ligands CD58 and ICAM-4 are critical for adhesion to NK cells via their receptors CD2 and aMB2, respectively. Interestingly, adhesion required CD16-dependent signals, but NK cells were also able to discriminate between infected and uninfected red blood cells even in the presence of polyclonal rabbit anti-RBC IgG. The authors then show that NK-mediated ADCC first resulted in damage to the IRBC plasma membrane followed by release of intact PV, and that these PV could be subsequently opsonized by immune IgG and phagocytosed by monocytes. Overall I found the manuscript to be fascinating, with many thoughtful experiments describing these important mechanisms of action of NK cells. I do have a few comments below that if addressed will assist in interpretation of the study findings.

Reviewer #2: Sekar et al. have in this manuscript aimed to study how NK cells provide protection during malaria. They do this by identifying key receptor interactions between NK cells and iRBC and mechanisms for NK-mediated iRBC damage/killing. They have further assessed the role of antibodies in this process and how those antibodies work together with monocytes to clear opsonized parasite material. To obtain this data they have used elegant in vitro systems and tools to visualize and measure the different processes. Overall, the manuscript is well-written and comprehensive and presents novel relevant data.

We thank both reviewers for the kind comments.

Part II – Major Issues: Key Experiments Required for Acceptance
Please use this section to detail the key new experiments or modifications of existing experiments that should be absolutely required to validate study conclusions.

Generally, there should be no more than 3 such required experiments or major modifications for a "Major Revision" recommendation. If more than 3 experiments are necessary to validate the study conclusions, then you are encouraged to recommend "Reject".

Reviewer #1: - My only major comment is regarding the specificity for the interaction/conjugate formation between infected and uninfected RBCs. In Figure 1a-C, the authors show that, in the presence of polyclonal anti-RBC IgG, NK cells only form conjugates with iRBC and not uninfected RBCs. This result is surprising to me. The authors speculate in the discussion that iRBC may be more rigid, favoring NK cell binding; alternatively, that the distribution of ICAM-4 might be different on iRBC vs. uninfected RBC and that this could favor aMB2 binding. Did the authors compare ICAM-4 expression between uninfected and infected

RBCs? Is that something that could be shown? Alternatively, are the ligands bound by polyclonal antiRBC IgG serum different between infected and uninfected RBCs?

It is the deformability of uninfected erythrocytes that is unusual, rather than rigidity of other cells. We have rephrased the description of our results and discussion with that in mind. New references that support our point of view have been cited. The rigidity of infected erythrocytes is orchestrated by *P.f*. shortly after invasion (Maier et al 2008) and essential for parasite survival. We are not speculating about rigidity of iRBC (it is a fact) but make the point that rigidity (also referred to as stiffness), which is well established as a property that favors cell-cell or cell-substrate adhesion could well be the basis for the difference between NK-uninfected RBC adhesion (very low or negative) and the measurable adhesion of NK cell to iRBC. Experiments to demonstrate it with *P.f*. infected RBC would be very difficult. Rigidification of uninfected RBC would be artificial and so would the 'relaxing' of infected cells.

We have removed the discussion about ICAM-4 distribution, as it is only one among many parameters that could account for differences in adhesion. Rigidification of iRBC remains the dominant hypothesis.

There is no discernable difference in the staining of uninfected and infected RBC with the anti-RBC antibody. This is shown in new Figure S1A. As the anti-RBC antibody used was generated against uninfected RBC, it is probably not binding to *P.f*. antigens exposed at the RBC plasma membrane. Not much is known about the abundance of proteins at the RBC plasma membrane and how it changes after *P.f*. infection. From the NK CD16 point of view it is the number and density of exposed Fc domains that matter. Due to the very high sensitivity of CD16 signaling (comparable to that of the TCR) we used the rabbit anti-RBC IgG at 1 to 4 μg/ml, which is approximately a 2,500-10,000 fold dilution of serum IgG levels.

- Relatedly - In the discussion, 2nd paragraph, the authors state, "This selectivity occurs even when uninfected RBC ad iRBC are coated with the same polyclonal antiRBC IgG serum [6]." I could not find evidence for this in the Arora et al ELIFE paper. Should that reference be removed since this result seems specific to the present paper?

We have removed that statement from the Discussion. The selectivity is shown in our own data in Fig 1.

- In the forward genetic screening experiments, the authors noted that NK cell lysis of uninfected EJ cells opsonized with anti-RBC IgG serum was comparable to NK cell lysis of infected RBCs. I am a little puzzled by this given the above observation of the specificity of killing in the prior experiments to iRBCs. They did note some differences in this model (e.g., blocking of aMB2 did not abrogate lysis, although gene ablation of ICAM-4 did). Did the authors try infecting the EJ cells with parasites to see how this might impact expression of cell surface markers and/or NK cell lysis? If not, possibly include this in the discussion as a potential limitation?

Part of our response is already in our reply to the first major comment. EJ cells, even differentiated, are not deformable. The fact that they form conjugates with NK cells is likely due to their rigidity. Furthermore, proteomics of cell surface proteins on a differentiated erythroleukemia cell line showed a remarkably high match with protein abundance on erythrocytes (Kanjee et al 2017).

We were fortunate that differentiated EJ cells coated with anti-RBC antibody formed tight conjugates with NK cells (not shown) and were readily lysed by NK cells (shown in Fig. 1). This allowed us to use them as a model system to directly test the contribution of ICAM4 and CD58 to lysis by NK cells. Infection of differentiated EJ cells with parasite results in invasion and some development but is not efficient enough to be a feasible system to use with NK cells. We do not consider this a limitation as we first used 3D7 infected RBC to identify receptor-ligand interactions and then used EJ knockout cells to validate those findings.

Reviewer #2: No additional experiments are required but there are several questions regarding the experiments that need clarification. These are added in the next section (Part III-Minor issues).

Part III – Minor Issues: Editorial and Data Presentation Modifications
Please use this section for editorial suggestions as well as relatively minor modifications of existing data that would enhance clarity.

Reviewer #1: - In the results, the authors show data that the parasitophorous vacule is opsonized by antibodies. Could the authors speculate in the discussion what the targets for these antibodies are?

We have added this to the discussion regarding the potential targets of antibodies (new text bracketed with << >>):

<<During invasion by a merozoite the PV is formed by invagination of the erythrocyte plasma membrane (52), a process that includes selective sorting of proteins and lipids (53, 54). For example, stomatin is excluded from the PV while flotillin is included despite their common presence in detergent-resistant membrane domains of erythrocytes (55). Likewise, phosphatidylserine (PS) is included while phosphatidylinositol (4,5) diphosphate (PIP2) is not (53). CD58 is among proteins that are included in the PV membrane during merozoite invasion. Note, however, that the inner leaflet of the PVM represents what was facing out at the RBC plasma membrane. Therefore, the CD2-binding domain of CD58 is expected to be *inside* the PV (55).>>

Do the authors have any data that these antibodies might accumulate with increasing exposure to Pf? (e.g. any correlations with age?)

This has been addressed with new text (bracketed with << >>):

<<Although ligands for human IgG on PVM are not known, anything not normally exposed to circulating blood may induce an Ab response, and that includes cytosolic domains of human proteins internalized during *P.f*. merozoite invasion and *P.f*. proteins that span the PVM, such as EXP2. PV released from iRBC are clearly immunogenic, as the titer of PV-binding IgG from malaria-exposed individuals is far higher than IgG that binds to iRBC.>>

Given that the titer of IgG in Mali plasma that binds PV is about a 100-fold higher than in plasma of US controls, we expect that these antibodies are acquired over time with chronic exposure to malaria. A longitudinal study with clinical data and correlates of protection would be very interesting.

- The authors show that these opsonized PV are then phagocytosed by monocytes. Did the authors determine whether these opsonized PV could be destroyed by other mechanisms? (e.g., NK-mediated ADCC? ADCP by neutrophils?) I wasn't entirely sure why monocyte phagocytosis was speculated to be the preferred route of elimination of PV; clarification here or in the discussion would help the reader.

We are not inferring that phagocytosis by monocytes is the main pathway for removal of circulating PV. Our data does show that Mali IgG triggers phagocytosis by monocytes but not that it is exclusive. We have now mentioned in the text that other Fc receptor-mediated responses could contribute, such as neutrophils.

- it's not very clear how many cells were used in each assay; could be helpful detail

Cell numbers are now given in the Methods section.

Figures
• 1A:
• The authors could be more explicit with the opsonizing conditions in the figure legend; the only visual difference between opsonizing and non-opsonizing conditions is filled-in shapes, many of which overlap. Filled-in dots/squares could be labeled more informatively, like "iRBC + Ab" instead of just "iRBC."

Agreed. We have now labeled the filled symbols in Figure 1A as "iRBC + Ab" and "RBC + Ab"

• A brief description of the setup, markers, and determination of cell conjugates used in the two-color flow assay may add helpful contextual information to readers.

Figure Legend for 1A now includes the following sentence: "NK cells were labeled with Cell Tracker Green and target cells were labeled with eFluor 670. After the indicated times, conjugates were assessed by flow cytometry by gating on double-positive conjugates."

A detailed description of the conjugate assay along with a reference is provided in the Methods section under the heading “Conjugate Assays”

• 1B:
• What do the three colors represent - three different donors? What time post co-incubation were cell-cell conjugates quantified? (pls add to legend)

Yes, the three colors represent three different donors. The cell conjugates were quantified at 40 min and this information is included in the figure legend for 1B.

• 1E + 1F:
• Although it’s clear from the text that the experimental setup for 1F was the same as 1E aside from using differentiated EJ cells, I think that could be clearer in the figures since they look nearly identical side-by-side

We have now labeled Figure 1 E and 1F as “iRBC” and “EJ-Cas9” respectively.

• 1G + 1H:
• Could the authors include wild-type / non Cas9-transfected EJ cells in these figures?

The standard control for CRISPR-Cas9 knockouts is the parental Cas9 transfected cell. Given the limitation in obtaining fresh, resting NK cells and the need to time each experiment to have differentiated EJ cells ready at the right time, we could not include a second control. The window of opportunity to perform such experiments was only a few days.

Figure 8
• 8B:
• The text specifies that primary monocytes and NK cells were used from multiple different donors: was each experiment done using cells from autologous donors?

Yes, each experiment was done using NK and monocytes freshly isolated from the same donor.

Would clarify that these donors were malaria-naïve since plasma is from malaria-exposed

We state that these donors are malaria-naïve on p.15.

Reviewer #2: Minor questions, comments, and clarifications asked for.

1) Is there a reason the NK cell conjugate assay (Figure 1A-C) does not work with the receptor blocks or why those experiments were not added?

Only a small proportion of NK cells form conjugates with iRBC, and yet the assay is very reliable (Figure 1C). However, due to the low frequency of NK cells in conjugates, this assay is not well

suited for inhibition experiments, such as antibody blocking. We preferred hemoglobin release as a reliable and meaningful assay for inhibition experiments.

2) It is interesting that so much of the NK-mediated killing remains despite blocking the ICAM4 and CD58 receptor-ligand interactions. Do the authors think there could be additional receptor-ligand pairs or is the remaining interaction by CD16-Ab alone?

There may well be additional receptor-ligand interactions that contribute to NK-mediated killing. We tested several other potential interactions but obtained negative data, which don't rule anything out. Overall, our data reveals two interactions that clearly contribute to lysis of iRBC by NK cells.

3) Although using the rabbit-anti-RBC standardizes coating of RBC, independent of parasites, it also leaves me wondering a bit how anti-RBC + iRBC + NK cells would correlate with for example Mali plasma + iRBC + NK cells. In figure 7 it seems there is very little binding of Mali IgG to iRBC, so would the NK cells in vivo be nearly as active/functional as what is shown here? Could the much larger RBC coating when using anti-RBC Abs influence for example the killing/breaking mechanism of the RBCs.

Mali IgG works at very high concentrations, in the > 1 mg/ml range. It is therefore too limiting for the type of study done here. The rabbit anti-RBC IgG was used in the 1 to 4 ug (micro-g)/ml range, which is sufficient given that engagement of CD16 is very sensitive and has a low threshold for activation.

4) Another Q following on the previous question is that by using rabbit anti-RBC, is there any influence on Fc-mediated functions compared to if human antibodies would have been used?

It is obviously not the same as human IgG, but rabbit IgG has been used reliably over many years as a ligand for human CD16.

5) The monocyte uptake assay is nice and the data is convincing. However, in blood the majority of cells would be neutrophils, which also express high levels of CD16 and are known to both phagocytose and use other mechanisms for killing pathogens and are proposed to play an important role (e.g. PMID: 34413459). Any thoughts on their role in this system/process? Perhaps add a line of discussion/limitation.

We don't claim exclusivity of monocytes. The potential role of neutrophils is now mentioned, and paper cited. Thank you for bringing it to our attention.

6) The results are mainly discussed in the context of adaptive NK cells and touch upon CD16low NK cells. But in the assays, total NK cells (CD56+) were used.

We discussed the results in the context of adaptive NK cells, because they are a prominent feature of people who have acquired clinical immunity to malaria, as we have reported (Hart et

al 2019). However, most US blood donors have very few adaptive NK cells. It was therefore not feasible to test them. There is no reason to think that adaptive NK cells, which have a strong intrinsic ADCC activity and elevated expression of CD2, would not perform well in the same experiments.

Minor comments
-Page 9 line 8: cells is written twice “cells cells”
-Page 12: There are two “is is” following each other.
-In figure 2B, it says Phalloidin 450, should be 405.
These typos have been corrected.

-When discussing hemozoin and its stimulation, it could be worth referencing this paper: PMID: 17261807.
Thank you for this suggestion. This paper has been cited.

-When introducing/discussing tolerance antibody-monocyte interplay, it could be worth to include this paper: PMID: 35443186
Another very nice study, now cited. Thank you for the suggestion.

PLOS authors have the option to publish the peer review history of their article (what does this mean?). If published, this will include your full peer review and any attached files.

If you choose “no”, your identity will remain anonymous but your review may still be made public.

Do you want your identity to be public for this peer review? For information about this choice, including consent withdrawal, please see our Privacy Policy.
Reviewer #1: No
Reviewer #2: No

Figure Files:
While revising your submission, please upload your figure files to the Preflight Analysis and Conversion Engine (PACE) digital diagnostic tool, https://pacev2.apexcovantage.com. PACE helps ensure that figures meet PLOS requirements. To use PACE, you must first register as a user. Then, login and navigate to the UPLOAD tab, where you will find detailed instructions on how to use the tool. If you encounter any issues or have any questions when using PACE, please email us at figures@plos.org.
Data Requirements:
Please note that, as a condition of publication, PLOS' data policy requires that you make available all data used to draw the conclusions outlined in your manuscript. Data must be

deposited in an appropriate repository, included within the body of the manuscript, or uploaded as supporting information. This includes all numerical values that were used to generate graphs, histograms etc.. For an example see here on PLOS Biology: http://www.plosbiology.org/article/info%3Adoi%2F10.1371%2Fjournal.pbio.1001908#s5.

Reproducibility:

To enhance the reproducibility of your results, we recommend that you deposit your laboratory protocols in protocols.io, where a protocol can be assigned its own identifier (DOI) such that it can be cited independently in the future. Additionally, PLOS ONE offers an option to publish peer-reviewed clinical study protocols. Read more information on sharing protocols at https://plos.org/protocols?utm_medium=editorial-email&utm_source=authorletters&utm_campaign=protocols
